# Footballers’ citizenship during COVID-19: A case study of Premier League players’ community support

**DOI:** 10.1177/10126902211045679

**Published:** 2022-09

**Authors:** Dr Charlie V L Smith

**Affiliations:** University of Leicester, School of Business, Brookfield, 266 London Road, Leicester, LE21RQ

**Keywords:** athlete citizenship, COVID-19, professional footballers, soccer, community

## Abstract

This paper demonstrates the community support of Premier League football players during the first COVID-19 national lockdown in the United Kingdom (March to May 2020). Given the global popularity and influence of footballers’ behaviour, it shows that they play an important role as citizens in supporting wider communities during circumstances such as the COVID-19 pandemic. A content analysis of 376 Premier League football players (80% of those registered) comprising 3877 posts on Instagram and Twitter is presented. The findings show 12 athlete citizenship roles during the pandemic which collectively illustrates players fostering support for fans and citizen's public health compliance, wellbeing and lives. Players also conveyed how they coped with the pandemic with their athlete mindset and were hopeful for a better future. The discussion and conclusion suggest that COVID-19 has presented an opportune time to renegotiate the complex social systems of which athletes are a part, identifying how they can engage in citizenship and future community support embracing the fullest range of their sporting profession.

## Introduction

Sport creates celebrities through its recognition of athletes’ specific accomplishments (Malcolm, 2012, Rojek, 2006). One of the primary characteristics of the sports celebrity has been with broader social and moral responsibilities that promote national pride and their qualities (Whannel, 2002). As the United Kingdom's (UK) national sport, football players are known for their physical skills, but equally their behaviour, personalities, and lifestyles off the pitch (Giulianotti, 1999, Vergeer and Mulder, 2019). Footballers and football clubs have been integral in their communities since 1975 when schemes were introduced to counteract the negative effects of hooliganism (Walters and Chadwick, 2009, Watson, 2000). Today football clubs still have an eminent local welfare function with all English Premier League teams having an established welfare mission statement and relationship with their local community (Kennedy and Kennedy, 2021). Supporting a football team is an important part of peoples’ lives globally, with fans often viewing players as their role models and heroes (Roderick, 2006, Rojek, 2006, Vergeer and Mulder, 2019). Such is the emotional attachment from fans that they often follow footballers and their lives as parables of normative behaviour, influencing how they live their everyday lives (Rojek, 2006).

During COVID-19 and its prolonged quarantine, human values have been critical in securing behavioural compliance with government guidelines and promoting prosocial behaviours to alleviate the pandemic's effects (Wolf et al., 2020). Whilst professional athletes often have fewer comorbidities than the general population, with a lower risk for severe disease or death, they are integral in preventing COVID-19 transmission and ensuring global health care systems do not exceed capacity (Toresdahl and Asif, 2020). Media frequently focuses on the entitlement of rich sports stars and the public's perception of their behaviour. During COVID-19 there has been significant media attention on athletes’ behaviour which has called for them to review their supportive role in society (Leng and Phua, 2020). For example, footballers have been accused of being greedy and ‘not pulling their weight in the crisis’ (Moore, 2021). There have also been cases of footballers’ inappropriate behaviours documented, such as Mason Mount (Chelsea) and Jack Grealish (Aston Villa) who were discovered breaching self-isolation guidelines (Leng and Phua, 2020). Beyond the individual level, whole football clubs have also stretched the guidelines on legitimate behaviour and travel. For example, Celtic embarked on their annual training trip to Dubai in January 2021 when the more virulent strain was identified in the UK and Scotland was under the highest restrictions. The players were also caught breaching social-distancing guidelines when their trip was already unpopular with fans and the first minister (BBC, 2021). Whilst the few examples of players’ breaches of lockdown rules received substantial public outcry, many have supported the community whilst fully abiding by the rules (Moore, 2021). Perhaps the most publicised example of a footballer's community efforts is Marcus Rashford (Manchester United) who campaigned for food vouchers for England's poorest families. 150 Premier League players also launched a collective initiative on social media named #PlayersTogether (BBC, 2020). #PlayersTogether set up ‘a contribution fund that can be used to distribute money to where it's needed most in this COVID-19 crisis, helping those fighting for us on the NHS frontline as well as other key areas of need’ (Players Football Association, 2020). Football players in professional leagues have also taken pay cuts of 10–30% following competitive matches being suspended (Evans et al., 2020). During the pandemic, and in athlete citizenship research thus far, the focus, however, has predominantly been on players’ monetary donations, rather than on other supporting acts that are more extensive, direct and available for broader beneficiaries. For example, of particular importance during isolation periods is fostering social connectedness within communities who may be feeling alienated, lonely and without meaning or purpose (Hoye et al., 2015). Drawing on the notion that athletes influence social awareness and change, and football's global popularity and influence, this paper identifies Premier League players’ citizenship during the unprecedented isolating moments of COVID-19. Doing this illustrates and contributes a more athlete-centred approach towards supporting community wellbeing and future understandings of athlete citizenship. The paper begins by outlining key ideas on sport, community and social capital and reviewing limited work on individual level athlete citizenship.

## Sport, community and social capital

The term community broadly implies democratic legitimacy, citizenship and representation of civil society (Jarvie, 2017). In association, sport has long been connected with community building, social welfare and fostering social capital to improve civil society (Jarvie, 2017). Whilst research has identified the relationship between sport and social capital, the collective premise of this is that social capital is an investment in social relations with expected returns (Widdop et al., 2014). These returns include those features of social life that enable beneficiaries to act together more effectively and achieve shared objectives, for example, taking collective action to reduce COVID-19's spread. The broader idea of citizenship in society is transferable from one human being to another, outside of contractual relationships and is transferred through empathy, affectional identification and emotional expression from one to the other (Hariman and Lucaites, 2001).

As Walker and Parent (2010) have summarised, the vast majority of sport organisation's websites reveal their participation in community outreach supporting social involvement with activities including giving, activating and capacity building. Specifically, these activities include tangible aspects such as monetary donations, supporting fundraising, in-kind donations, volunteering and emotional aspects like public service announcements (Inoue and Havard, 2015, Kim and Walker, 2013). Research on these initiatives predominantly highlights the strategic and corporate benefits that a football club can gain from their implementation through a community trust model of governance (Walters and Chadwick, 2009) and often which are determined by stakeholders’ demands. The overriding stance of these initiatives is ‘communitarianism’ which concerns developing and preserving the community's wellbeing (Kennedy and Kennedy, 2021). Sport is an ideal example of the mutuality between political and social theory and practical politics (Jarvie, 2017) and especially when members of the community are attached to a sporting ideal or common objective (Jarvie, 2017). In sport, there is also a close connection with public health whereby athletes are citizens of the wider community in which they belong, and which is comprised of other athletes, support teams, families, and local, national and international societies (Mann et al., 2020).

## Athlete citizenship

Whilst athletes remain citizens of broader communities, citizenship per se exploring their behaviours at the micro-level have had sparse attention beyond focusing on monetary donations and philanthropy as reflected in corporate social responsibility (CSR) literature. Exploring only monetary initiatives is a reductive view of the athlete, negating their other traits, especially those which are more personable and those which may derive from the true spirit of their life and work. Agyemang (2014, 26) has defined athlete citizenship ‘…as the manner in which a professional athlete conducts himself or herself (on and away from competition) and makes a positive impact on society’. Thus far, a key purpose of athletes’ citizenship research noted has been eliminating resentment that the public has of them, often linked to money (Agyemang, 2014) and occasionally off-field instances of misbehaviour (Osborne et al., 2016), rather than focusing on other acts of kindness that might derive from events such as a global pandemic. Critcher's (1979) seminal work has also traced the historical trajectory of football players since the war and shows that the dramatic change in their economic situation demonstrated their shift from being originally connected to working-class communities towards becoming estranged superstars and heroes.

Using social media athletes can shape society's impression of them, while simultaneously letting them provide open accounts of their lives with fans, friends, and family members (Shreffler et al., 2016). Sauder and Blaszka (2018) show this when exploring the United States’ women's national soccer team's content on Twitter during the 2015 World Cup, identifying six backstage and four frontstage frames of players’ interaction. The backstage frames were denoted as *conversationalist* (direct interaction with athletes, celebrities, family and friends), *sports insider* (behind the scenes view of their football lives), *behind the scenes reporter* (candid reports of football player's persona), *super fan* (discussion of non-football sport), *informer* (general information sharer) and *analyst* (general statements of opinions, complaints and musings). The front stage comprised *publicist* (player's promotion with aspects such as upcoming matches), *superintendent* (maintaining the player's promotion), *fan aficionado* (direct interaction between the player and fans) and *brand manager* (formal acknowledgement associated with a positive image). Similarly, Smith and Sanderson (2015) examined the captions used by athletes with their Instagram photos and identified 6 roles including humanitarian, family-driven, personality traits and interests, dedicated athlete, endorser and socialite.

The empirical work herein explores how footballers as globally prominent and popular athletes have helped during the pandemic and identifies 12 roles of citizenship in their community support. By identifying a range of initiatives beyond monetary support, the research develops the concept and detail of athlete citizenship at the micro individual level and encompasses a more extensive range of behaviours that identifies athletes contributing to community work in the spirit of their demeanour.

## Methods

The study is a content analysis identifying core consistencies and meanings from Premier League footballers’ Twitter and Instagram posts during COVID-19. The Premier League is the biggest league in Europe with a global representation of player's nationalities. Online data collection of players’ Instagram and Twitter accounts was suitable for immediacy as they often posted their thoughts and feelings during COVID-19. Proliferating social media also means fans can easily receive insight into the personal and professional lives of their favoured players (Hayes, 2020). Players’ posts expressed their thoughts, feelings and emotions during COVID-19 and displayed their community-facing support initiatives. The dataset comprised 376 registered Premier League players who had either Tweeted and/or posted on Instagram between the 13^th^ of March 2020 (when matches were suspended) and the 23^rd^ of June 2020 (when all teams had played their first fixture post national lockdown). The UK has subsequently had further lockdown periods since the data collection period, however, in later times players continued their roles as professional footballers. In total 376 (80%) out of 469 officially registered Premier League players had a qualifying post for the analysis on either platform. There were 244 players solely posting on Twitter, 132 players just posting on Instagram, and 332 posting on both platforms. Each post was screenshot and then directly imported into the qualitative analysis software, NVivo. Most players posted a photo or caption, but when they included a video, the overall caption and its first frame was imported for analysis. Only players’ official and verified accounts were included. Fans and team's accounts were excluded because the focus was on individual players as citizens posting by themselves, and not those being prompted in line with their club or sponsor's contractual agreement. Any paid partnerships were labelled as such by both platforms and excluded. Theoretically, athletes are thus framed as self-governing individuals who choose and control their posts (Sauder and Blaszka, 2018). The analysis only included original posts of players during the specified period and excluded replies or retweets which could not have been taken as an endorsement. Furthermore, it was unnecessary to only select posts with explicit mention of COVID-19 as the crisis was so encompassing that virtually every post mentioned it and thus such was an unviable inclusion or exclusion criteria.

### Data analysis

After importing the entire corpus into NVivo, the software was used to view and scan through each post individually in an inductive fashion before determining a set of codes for application throughout, with each Tweet or Instagram post considered as a single unit of analysis. This familiarisation process involved repeatedly reading/viewing all the data to obtain immersion and sense of the entire corpus. The approach was a more conventional content analysis that avoided using preconceived categories (Hsieh and Shannon, 2005) and was necessary given the paucity of theorisation on athlete citizenship. When producing codes, the accompanying image and any activities (and objects) occurring in it were particularly important, as was any accompanying text written by the player expanding on their post's intention. After initial coding, the posts were worked through again looking specifically at two enquiries that became apparent and matched key UK public health and political discourse during COVID-19; whether people in pictures appeared socially distant and whether the hashtags #staysafe or #stayhome were used. Whilst global discussion ensues around the safest distance between people, posts were coded in this way if the player was alone or appeared circa two metres distance from others pictured, indicating implicitly or explicitly their intentional distance. The picture taker's identity was rarely stated, but they did not appear as ‘selfies’, which perhaps queries social distancing if they were not taken by those they were ‘bubbling’ with. The objective of the analysis was to determine what players were doing, who they were doing it with/for and the overall message their post conveyed. The post's date gave additional insight into the lockdown stage and further clarified some of the implicit messages made. For example, if a post was posted after the 23^rd^ of March but before the 20^th^ of May then this was likely taken when the UK was in national lockdown but before players returned to training. As part of the presentation and discussion of the data, exemplary posts are quoted reflecting the data set's key trends. The frequency of themes and codes are shown to note their prevalence.

## Findings

The majority of posts included a picture, with 86% (n = 3336) of these pictures being of players themselves, including when they were referring to someone else. Whilst perhaps narcissistic, this showed the basis and integral part of communication with fans, with players showing them that they are still there, keeping in touch and offering support during COVID-19. The proceeding narrative in the findings shows how Premier League footballers lived and behaved as citizens during the first COVID-19 lockdown, adopting 12 different roles, overviewed in Table 1. There were 4801 codes applied across the dataset, and the number of occurrences seen in the table below shows the proportion of the codes that each role represented, building a profile of citizenship activities and support initiatives that footballers engaged in.

**Table 1.** Overview of footballers’ citizenship roles during COVID-19.

Footballers’ role	Definition	Number of occurrences	Examples
Humanitarian carer	A player concerned with and/or seeking to promote human welfare throughout the community	470 posts 9.79%	Donations to key workers Community phone calls Fundraising community challenges Sending best wishes
Joker humourist	A player joking with acts of trying to please or amuse, often about COVID-19	312 posts 6.50%	No haircuts Playing football socially distant
Dedicated athlete	A player motivated in achieving their highest performance goals physically and mentally despite COVID-19	690 posts 14.37%	Home training and its struggles Sharing workouts Motivational quotes
Everyday struggler	A player referring to struggling with the reality of everyday life during COVID-19	131 posts 2.73%	Boredom Unable to buy essentials Mental health
Reminiscer and reflector	A player who mentions and indulges in an enjoyable recollection of past events during COVID-19	512 posts 10.66%	Winning promotion or other competitions Missing the game Throwbacks to football beginnings Favourite games and goals
Family man	A player showing their family and home life activities during COVID-19	356 posts 7.42%	Spending time with family including whilst training Playing and bonding with children
Celebrator	A player celebrating or recognising a special event during COVID-19	176 posts 3.67%	Easter Mother's Day Father's Day Birthdays Anniversaries
Religion follower	A player showing faith in something and that COVID-19 will improve	97 posts 2.02%	Praying COVID-19 gets better Religious quotes
Rule endorser	A player advocating their role as a citizen in conjunction with politics and their principles during COVID-19	207 posts 4.31%	#StayHome messages Demonstrating face covering, hand washing and social distancing Anger at non-compliance
Socialiser	A player mixing socially with others or who plays a prominent role in high society and invites socialisation from others online during COVID-19	308 posts 6.42%	Asking fan's opinions Holding live chats
Hope spreader	A player giving support, confidence or hope to others during COVID-19	1256 posts 26.16%	Reminding fans football will return soon Hoping COVID-19 situation improves
Hobbyist	A playing sharing their particular hobbies, both old and new outside of football	286 posts 5.96%	Playing e-games Music Household chores Reading Television

## Dedicated athlete

During the pandemic home workouts became the new normal for a considerable part of the population. Social media was significant in disseminating virtual fitness with notable figures, celebrities and athletes sharing their knowledge (Hayes, 2020). One of the most popular in the UK was Joe Wicks, ‘The Body Coach’, who attracted millions of viewers with his free online workouts. Similarly, the pandemic presented the opportunity for players to share examples of their training for others to try. 14.37% (n = 690) of the posts demonstrated players’ training or their athlete mindset they adopted to help them deal with the pandemic's challenges. Professional footballers train year long, but usually with others and rarely alone. The findings show that players’ posts resonated with the rest of society who also had to exercise at home during COVID-19. Alex McCarthy (Southampton) posted ‘A look into my training routine at home’. They also shared their struggles, Josh Brownhill (Burnley) posted upon beating his best running time ‘Some days are harder than others during this time to keep motivated but today was a good day!’. Players also detailed the extra efforts they were making to maintain their fitness. Some of the most motivated and disciplined posts came from Odio Jude Ighalo (Manchester United) ‘We don’t stop when we are tired, we stop when we are done’.

Players frequently posted from their home gym, and whilst these facilities are unaffordable for many fans, they were often ‘make do’ adaptations. Fede Fernandez (Newcastle United), trained in a small space, near his back door, with limited equipment and surrounded by kids’ toys. Angel Ogbonna (West Ham United) ran in a dark car park ‘Out of the pitch…into the struggle #runathome #training’. Players’ sporting mindset extended across the circumstances of the pandemic and beyond their training. Trezeguet (Aston Villa) posted a picture of himself in a previous match keeping the ball from Cristiano Ronaldo ‘Never give up, great things take time. Be patient’.

## Everyday struggler

A small number, 2.73% of posts, n = 131 referred to struggling, accompanied by a serious side to mental health and wellbeing. Michail Antonio (West Ham United) posted a black and white picture of himself:

One thing we should all never stop working on is our mental health. Best way to start is to get talking about it. Lockdown has been a challenge for a lot of people mentally, something that can help is to share how you’re feeling.

Steve Cook (AFC Bournemouth) ‘I’ll be on the end of the phone, if you are struggling do not hesitate to get in contact with the club’. Gini Wijnaldum (Liverpool) posted dressed in black, ‘Current mood…some things are bigger than football. Take good care of yourselves and your loved ones’.

Players’ mental health difficulties were also accompanied by boredom, something readily felt across the population. Allan Saint-Maximin (Newcastle United) joked about ‘#stayinghomefor #day350’ early in the lockdown. Players also mentioned being unable to get a haircut, the difficulties of caring for homebound children and sourcing essential supplies dues to panic buying. There were also posts about players’ daily routines, many sharing a day of their life in lockdown with their family. Pablo (Arsenal) shared ‘Hi everyone! I hope you are well. This is how I’m spending my time at home with my family’.

## Hobbyist

With more free time, players discussed their hobbies and alternative activities whilst staying home. There were n = 286 posts (5.96%) referencing hobbies apart from football. A popular hobby was playing football computer games and sharing competitions (e.g. Nicolas Otamendi, Manchester City). Other indoor activities included playing musical instruments (e.g. Nathan Ake, Bournemouth), sharing music playlists (e.g. Ben Osborn, Sheffield United), watching Netflix (e.g. Jeff Hendrick, Newcastle United), gardening (e.g. Erik Pieters, Tottenham Hotspur), household chores (e.g. Tammy Abraham, Chelsea), dog walking (e.g. Raul Jimenez, Wolverhampton Wanderers), reading (e.g. Adrian, Liverpool) and cooking/baking (e.g. Oriol Romeu, Southampton). Partaking in other sports was also popular, such as cycling (e.g. Ben Foster, Watford), boxing (e.g. Ben Godfrey, Norwich City), playing golf (e.g. Paul Dummett, Newcastle United) or mentioning other sports they watched on television.

## Family man

There were n = 356 (7.42%) posts documenting players spending time with their family, often stressing this was more important than football. Shkodran Mustafi (Arsenal) epitomised this sentiment of family life when sitting with his toddlers by a duck pond ‘Of all the titles I’ve been privileged to have, “Dad” has always been the best’. Players regularly referred to Mothers’ and Fathers’ day that occurred during the lockdown. Jordan Pickford (Everton) posted ‘Happy Mother's day to the most amazing and caring mum to our boyo, we love you so much’. Wilfred Ndidi (Leicester City) posted ‘Happy Father's Day’ holding his new-born daughter.

Players posted how their families had become interspersed with their football lives and routines during COVID-19. For example, Harry Maguire (Manchester United) sitting with his daughter ‘Eat. Sleep. Train. Peppa Pig. Repeat’. Players also used cohabiting family members to support their training, Tomas Soucek (West Ham United), playing football with his wife at home ‘The fun is at home too #stayathome #football #family #home #fun’. Players also demonstrated their adaptations by showing how they continued training with their kids at home. Andy Carroll (Newcastle United) did squats holding his daughter ‘Squat and throw wolf #GymDone’. Kevin de Bruyne (Manchester City) ‘Creative leg day with the kids’ with his twins on his legs whilst lifting them like a leg extension. There were also multiple posts about home-schooling. Roberto Firmino (Liverpool) illustrated sitting with his two children at a desk, as did Jamie Vardy (Leicester City). Players also provided activities to help school kids’ learning. David Luiz (Arsenal) being one and Bjorn Engels (Aston Villa) being another who presented a ‘learn Flemish’ video. Notably few players mentioned being separated from their extended families during the lockdown. A rare example was Dominic Calvert-Lewin (Everton) ‘Missing my little brother at the minute, don’t take this family time for granted’.

## Celebrator

Traditional celebratory dates in player's and national calendars were recognised but they were celebrated differently during the quarantine. A list of national events occurring during the UK lockdown can be seen in Table 2, and n = 176 posts (3.67%) mentioned these. The majority referred to Easter Sunday, Ramadan and Victory in Europe day (VE day). Gini Wijnaldum (Liverpool), with a picture of him wearing his orange national colours wrote ‘Happy Kingsday Holland! Hope you can find ways to enjoy yourself today #staysafe #Koningsdag2020’. Birthdays featured highly, Florin Andone (Brighton & Hove Albion) shared a picture of a cake with 27 candles, with his Mum, girlfriend and dog:

**Table 2.** List of events during the period studied.

Event	Date
Premier League matches suspended	13^th^ March 2020
Government informs all unnecessary social contact should cease	16^th^ March 2020
St Patrick's Day	17^th^ March 2020
Mother's Day (UK)	22^nd^ March 2020
Beginning of UK national lockdown	23^rd^ March 2020
Easter Sunday	12^th^ April 2020
St George's Day	23^rd^ April 2020
Victory in Europe day (VE day)	8^th^ May 2020
Mental health week	19^th^ May 2020
Return to training (various stages)	20^th^ May 2020
Eid Mubarak	23^rd^ May 2020
Black Lives Matter protests	Since 28^th^ May 2020
Father's Day (UK)	21^st^ June 2020
First competitive matches of the return (behind closed doors)	21^st^ until 23^rd^ June

Thank you very much for all your messages for my birthday, really appreciate it. Different birthday but doesn’t matter, I’m really blessed to be healthy and have my family with me. Wish you also all the best in this difficult situation and hope to see you soon.

## Religion follower

Some of the celebrations and festivals mentioned above were religious events but n = 97 posts (2.02%) displayed additional account of religion as a means of offering hope, a theme explored in later findings below. Marvelous Nakamba (Aston Villa) posted in the sunshine, alone, dressed in bright colours, looking up to the sky ‘I will always look up to God and I will forever be thankful to him. I hope you’re all well and have a blessed week’. Following a faith also helped athletes understand the situation, Domingos Quina (Watford) ‘God has a plan. Trust it, live it and enjoy it’. Another example was Divock Origi (Liverpool) ‘God be with us, in these times. My prayers go out to the world. It's time to fight this virus, kill all fear and spread positive energy. Bless!’. These posts showed how players felt helpless and looked for further sources of faith.

## Socialiser

There were n = 308 (6.42%) posts where athletes directly asked fans for their engagement. They also asked for engagement from other football players using the at (@) symbol. Players’ attempts at facilitating socialising were apparent with rhetorical questions and question marks in their posts. Players used this engagement as a time filler, getting closer to fans, often asking ‘What do you think?’. Theo Walcott (Everton), doing the hoovering, in tracksuit bottoms and a glass of wine in his hand wrote ‘How's everyone's Saturday night? #lockdownsaturdays #stayhome #savelives’. With thousands of social media followers, players were unlikely seeking fan's opinions or could they engage with every post Instead, they were providing virtual space that gave fans something to do during the enforced social restrictions.

Further to soliciting opinions, players also shared various activities for fans to partake in. For example, they frequently held Instagram live chats and later in the pandemic when some quarantine rules had been released gave fans the chance to win a training session with them. Aaron Connolly (Brighton & Hove Albion), for example:

Stay at home giveaway! To win this signed shirt, all you have to do is tag me in your Instagram story with your favourite skills or celebration (which I will do for my next goal) and I will announce the winner on my Instagram story on Wednesday #BHAFC #Giveaway.

Chris Wood (Burnley) ‘Thanks @Dannyboyr4 if you are a football person please join the challenge of posting a football photo. Once nominated you have 24hrs to post a picture of yourself as a player or donate £30 to charity & tag 4’. Billy Gilmour (Chelsea) ‘I’ll be streaming on the @CombatCorona FIFA 20 charity stream with other professional footballers to raise funds to combat #Coronavirus. Follow @CombatCorona for more!’.

## Joker and humourist

Despite the globally threatened public health, n = 312 posts (6.50%) included humour. Apart from these posts’ tone, joking was apparent through players’ inclusion of the laughing emoji and humour was used to acknowledge the exceptional and bizarre times. Players most often joked about the lockdown and conveyed their message with cartoons. Many players presented their portrait with their family at home in the style of the tv programme *The Simpsons*. Paulo Gazzaniga (Tottenham Hotspur) did this and added the caption #UltimateQuaranTeam. The use of the word ‘team’ emphasising that his team is now his family at home and not his professional football team.

Other jokes concerned social distancing in the football setting once players returned after lockdown. Fred Guilbert (Aston Villa) ‘Oh @UEFA.com-fr I found the solution to start the season again’ upon posting pictures of individuals in large socially distant bubbles with a small football nearby. Tyrone Mings (Aston Villa) posted himself at training in early May ‘Good to be back training with all my mates’ when the picture depicted him alone. Commonly players also joked about previous times before social distancing was needed, Billy Sharp (Sheffield United) ‘1 year ago @Sheffield United, magical moments, no social distancing on this day’. Given the level of conformity otherwise illustrated with players’ social distancing, these jokes appeared as means of coping with the new enforced way of life and for fans’ entertainment. Haircuts were also commonly joked about. Ezequiel Schelotto (Brighton & Hove Albion) posted a picture of his wife cutting off his ponytail. Sead Kolasinac (Arsenal) posted a cartoon picture of his long and unruly hair whilst in the gym, ‘No barber, no problem! Even when you look like me at the moment…it's important to stay home!’.

## Humanitarian carer

Players’ posts showed care for members of society (n = 470 posts, 9.79%). As mentioned already, during the lockdown the most publicly spoken about player's efforts was probably Marcus Rashford (Manchester United) who campaigned to make food vouchers available for children. Players also mentioned frequently donating to key workers. Christian Pulisic (Chelsea) ‘To show my gratitude to the superheroes in my hometown, Hershey, PA – myself and @Chipotletweets will be sending meals every Saturday (starting yesterday) for the next month to all the brave men & women fighting COVID-19 @PennStHershey’. Players also posted support with pictures of NHS workers imposed outside their club's stadium. James Justin (Leicester City) at 8.04pm on March 26^th^, 2020 ‘Hope everyone was paying their respects to the NHS workers and carers that are keeping everything together at the moment #ClapForNHS’. Other examples included Pedro Rodriguez (Chelsea) ‘An applause to all people that are working on helping others. Thanks a lot!’ and Yan Valery (Southampton), clapping at the end of a game alone: ‘A big thanks for all the @NHSuk staff’. This suggests players felt some degree of helpfulness and were grateful, much like the rest of society.

Players utilised their extensive social networks to encourage public donations. For example, Ben Gibson (Burnley) ‘Attempt 26 keep ups, nominate 5 people & donate £5 for Teeside Hospice because they’re currently fighting a battle & helping NHS’. Players offered emotional support too. Maty Ryan (Brighton & Hove Albion) posted:

Hoping you and your loved ones are managing to stay safe and healthy. Fingers crossed we can get control of the circumstance and can return to our normal way of lives in the not too distant future #stayhealthy.

Lastly, players offered individualised and personalised support publicly. For example, Pep Guardiola's (Manchester City manager) mother passed away during COVID-19 which led to multiple Manchester City players posting a picture of themselves, embraced with him.

### Black lives matter

George Floyd's death was mentioned prevalently by players from the 25^th^ of May 2020 and ‘Black Lives Matter’ was printed on their match kit. Using the power of their voices and position in society, their posts were a general symbol against racism but also motivated by decades of racial inequality and discrimination in English football (BBC, 2021). Patrick Van Aanholt's (Crystal Palace) words were exemplary:

As a player of a club in a community so diverse with culture, players, staff & fans, it's on all of us to speak up about those that look to keep us separated rather that united. I’m no politician but I know we must all play our part to help fight this evil #BlackLivesMatter.

## Rule follower and endorser

Citizenship assumes people's membership of a political body as part of a nation-state, with this unfolding relationship informing the enactment of guidance (Guschwan, 2014). Players reflected political discourse and guidance with n = 207 (4.31%) posts. Paul Pogba (Manchester United) referred to the World Health Organisation guidelines ‘We must all BE READY for #coronavirus follow @WHO advice to be ready’. Gary Cahill (Crystal Palace):

This is an urgent message. To help save lives, you must stay at home. Only leave your home to buy essential food, medicine or for individual exercise and always stay two metres away from other people. Stay home. Protect the @NHSuk Save lives #StayHomeSaveLives.

472 posts incorporated the hashtags #staysafe or #stayhome, a key UK government message for stopping the virus’ spread. Players emphasised these measures concerned the collective effort of everyone in society reducing the virus’ spread, and not just them individually. When they posted pictures of themselves, they exemplified substantive social distancing, with 1742 posts (47.3%) doing this.

When mentioning key events that players considered might deter the public's guidelines compliance, they incorporated the hashtag #StayHome and #StaySafe and often with the emoji of a house with a heart-shaped globe inside. For example, Andros Townsend (Crystal Palace) posted that individuals must stay home on Easter Sunday when people wanted to be with their families and another frequent example was when good weather could tempt people away from their homes. Other compliance to national guidelines included players sharing videos of handwashing for 30 s and advocating wearing face coverings (e.g. Newcastle United's Valentino Lazaro). James Milner (Liverpool) posted a picture of books he was signing, wearing gloves. ‘Sunday's safe signing session #cleanhands #askafootballer #LFC’. Players posted #StayHomeSaveLives challenges, giving fans a home-based activity as means of persuading them to stay there. Mezut Ozil (Arsenal):

To make sure you are really staying at home; I’m inviting you all to my #M10 jersey challenge: send me your pictures with my jerseys from home and I’ll share the best ones on my social media #StayHomeSaveLives.

Players were angry at people breaking the Covid rules. Aaron Connolly (Brighton & Hove Albion) ‘Why am I seeing stories on Instagram of people having a big party, they’ll be the first ones to complain about holidays and festivals being cancelled, it's just mad’. Jan Vertonghen (Tottenham Hotspur) ‘Should fine big time everyone who is not respecting the protection measures and give the money back to NHS, doctors, nurses, key workers etc’.

Players still emphasised maintaining safety measures when the lockdown was easing. When they returned to training grounds (circa 19^th^ May 2020), their posts reflected the government's football guidelines required, notably going already changed into their kit. Ilkay Gundogan (Manchester City) posted in his club kit, donned with mask and gloves ‘Off to work’. When players started returning to normal, they were aware the rest of the population remained under more stringent messages and responsibly encouraged them to maintain them.

## Hope spreader

Hope spreading constituted the largest amount of codes n = 1256 (26.16%) and was seen in two main ways. The first of these was the messages players conveyed themselves about getting through the virus safely. At the lockdown beginning Todd Cantwell (Norwich City), for example, posted ‘I now want to wish you all health and to be careful through this difficult time. See you all soon #oneclub #staystrong’. Another example, Andre Gray (Watford), who sat in a patch of sunshine, light reflecting off the wall behind him ‘Through tough times we’ve got to stick together, at the end of the tunnel there's always light’.

The second manifestation of hope was players giving fans something to look forward to. As part of this, players posted their happiness at returning to training, Pepe Reina (Aston Villa) posted ‘Happy to comeback’ with his fists pumped. Victor Wanyama (Tottenham Hotspur), on the 20^th^ of June, posted ‘The hard work continues’. When returning to matches, players counted down the days, emphasising that they would ‘Soon be back on top’ (Steven Bergin, Tottenham Hotspur) and fans would have something to enjoy. They also posted pictures of descending numbers on their shirts, increasing the immediacy and excitement for fans. This also reflected players’ dedicated athlete mindset. Tim Krul (Norwich City) airborne during a game ‘Another day closer to “normal”…#nevergiveup #countingdownthedays’.

## Reminiscer and reflector

Players posted about missing the game and their fans’ support, wanting to be doing their job, playing competitive matches. This represented 512 posts (10.66%). These posts included archived photos as they often contained images of full stadiums before social distancing requirements. Further to missing the game, the COVID-19 pause gave players specific time for reflection and posting happier memories. #ThrowbackThursdays were frequent. Players also remembered scoring their favourite goals, winning trophies or gaining promotion. Christian Fuchs (Leicester City) posted a picture lifting the Premier League title with the caption ‘Happy Anniversary! #5000 to 1’. Players also commonly posted about their footballing history and journey. Reece James (Chelsea) posted a younger version of himself ‘Just a little boy with a big dream’. Another example was Kasper Schmeichel (Leicester City) who posted a picture of his dad (Peter Schmeichel) laying on the grass, with himself kitted up similarly, holding a ball and standing beside him ‘A young boy with big dreams’. Sharing their journeys reflected the player's progress and was interpreted as a similar line of hope for COVID-19. It also provided memories of better times that could give fans temporary joyful moments away from the pandemic.

Posts were often made at 3 pm on a Saturday when traditionally players would be playing football. Upon their return to competitive action, when fans were prohibited in stadiums, players’ posts also included phrases such as #Apartbuttogether and ‘Empty stadium[s] aside’ (John Egan, Sheffield United). Isaac Hayden (Newcastle United) posted ‘We know you couldn’t be with us, but we felt the support in spirit’. Whilst supporting at home is second best for fans, players were highlighting their gratitude for both being supported and for the public still following guidelines and supporting from a distance to maintain society's greater good.

## Discussion and conclusions

Following a paucity of research on the role of athletes as citizens, this paper has analysed Premier League football players posts on Instagram and Twitter during COVID-19. The key contribution shows how players’ posts have offered parables to fans and the community of how they might live their lives during the pandemic, identifying 12 citizenship roles fostering public health compliance, wellbeing and lives. The findings overall also demonstrate how the pandemic has provided the space for players to show how their devoting athlete demeanour can be applied to broader social and public health concerns beyond monetary donations. Furthermore, the findings did not reflect literature that suggests athletes use social media for branding purposes and to boost earning potential (Choi and Berger, 2010). Notably, players’ community responses seen herein are rather more individualised than previous research that has documented the sanitized and impersonal communications that are disseminated through a club's public relations department as part of their CSR agenda. The paper now closes by surmising the key implications of these findings, before noting future research.

From the outset, players’ sporting mindset and athletic emotional makeup were apparent in how they dealt with, overcame, and helped others during COVID-19. Hope connected many of the citizenship roles identified as players shared their images, actions and aspirations (Adam, 2008) and which may have helped fans compensate for the pursuit of their life goals that were inevitably paused during the pandemic. Fundamentally players supported society by reminding them of happier sporting events and sharing their rejuvenated plans for football post-COVID-19. Players used the future as a realm of potential and possibility that could be filled with new dreams, desires and plans post-pandemic (Adam, 2008). Footballers also shared their personal lives publicly, presenting their everyday experiences and struggles with COVID-19, and thus challenged sporting discourses as players being strong, brave, and tough (Lines, 2001). Players also shared their private domain, such as their daily routines, their diets, how they were spending spare time and how they supported their children, as a means of helping fans through the routines of everyday isolating pandemic life (Rojek, 2006). These insights were a key part of demonstrating players’ existence in a welfare state outside of their lives as footballers, thereby being responsible and active citizens as part of the community during COVID-19 (Brown and Baker, 2012). Whilst elite football players are often presented as privileged members of society given their stardom and accrued benefits (Lines, 2001), this image has been challenged herein as players became more normalised members of society who faced the same struggles as fans. Players’ posts illustrated their pride in fulfilling national qualities and supporting the government's key safety mottos such as ‘stay home, save lives’.

For fans, seeing their football heroes and role models comply with COVID-19 rules likely encouraged an affinity that consequently enforced their own compliance of public health guidance (Wolf et al., 2020). Similar effects occurred with players encouraging citizens to exercise and take health precautions.

The citizenship roles of athletes identified could apply to many public health crises and future disasters, given their underpinning of players’ fundamental sense of being and dedicated mindset. Furthermore, given how engrained social media is within the sports industry, opportunities for athletes to continue this citizenship will likely be sustained post-pandemic (Hayes, 2020).

### Further research and limitations

Whilst the content analysis presented herein has shown the breadth of players’ citizenship work during COVID-19, the method is limited to it being a snapshot of players’ posts during the pandemic's height and thus does not include fans’ replies. Future research should use in-depth methods to explore how players connect with wider social media networks and how they engage with broader communities of interest in more detail. For example, it would be useful to measure and interpret fans’ engagement with posts. Furthermore, in football, gender-based norms and equalities remain (Evans et al., 2020) with the media focus being weighted towards the restoration of the men's professional sports leagues during COVID-19 (Rowe, 2020). Exploring female player's citizenship, undoubtedly, requires investigation, not least as their prevalence in football grows but also because their roles will likely present different emotional and lived experiences. Professional female footballers could not be included herein as so few posted during lockdown and comparisons would have been meaningless. In addition, since its inauguration, the Premier League has seen increasing diversity of national origins amongst players, in the 2018–2019 season only a third of players were English (Penn and Penn, 2020). Vergeer and Mulder (2019) have already suggested foreign players may have more of an impact online with those of the same ethnic origin (Vergeer and Mulder, 2019) and thus considering race and ethnicity's influence on the interactions may usefully indicate how players’ citizenship activities could be maximised.
